# Evolving COVID-19 conundrum and its impact

**DOI:** 10.1073/pnas.2007076117

**Published:** 2020-05-07

**Authors:** Thanat Chookajorn

**Affiliations:** ^a^Genomics and Evolutionary Medicine Unit, Centre of Excellence in Malaria Research, Faculty of Tropical Medicine, Mahidol University, Bangkok 10400, Thailand;; ^b^COVID-19 Network Investigations Alliance, Bangkok 10400, Thailand

Forster et al. (1) performed a phylogenetic network analysis with 160 severe acute respiratory syndrome coronavirus 2 (SARS-CoV-2) genomes from a public database and proposed a network of ancestral nodes and derived types, suggesting virus adaptability to specific geographical regions.

The median-joining network approach employed in Forster et al. (1) is a method made popular by its success in tracing the evolutionary history of human mitochondrial genes unresolved by conventional phylogenetic tree construction methods due to their small genetic distances within a large sample size (2). It measures the differences in sequences (Hamming distance) and determines median vectors of the network by relying on minimum spanning tree and maximum parsimony (3, 4). However, the question arises as to whether the median-joining method is suitable for the analysis of global pandemic SARS-CoV-2 that has just been introduced in humans on a global scale. An important determining factor is how the approach deals with the “missing data,” those from evolving viruses passed among infected individuals along undiagnosed transmission lines and not sampled for sequencing (5). Without them, the median-joining network method could still shape existing data points into groups sequentially linked together as a series of perceived transmission network events. The cascade of transmission routes from the ancestral node presented in the paper would be correct only when a sufficient number of cases are included in the network. Otherwise, the approach might generate a deduced network based on Hamming distance and a presumed cascade of mutational accumulations, leaving the conclusion uncorroborated by existing evidence. In fact, they are clustered within the lineages previously grouped by a maximum-likelihood method (6). It is tempting to assume that the A, B, and C groups were adapting to the local environment in specific geographical regions as the authors speculate. However, there is no concrete evidence supporting the functional significance underlying the measurable changes during the outbreaks, and new hypotheses associated with mutations undergoing lineage-specific fixation have to be experimentally proven (7).

As an evolutionary biologist working in a developing country, I have experienced firsthand how sensational findings can influence decision-making processes by diverting time and resources to control virus strains deemed to be “more aggressive.” In the fog of war, scarce resources are allocated in haste, and the developing world does not have well-informed science advisers sitting in every key meeting to help provide balanced scientific viewpoints. The scientific community, as a whole, needs to be extra cautious in interpreting new findings related to coronavirus disease 2019 (COVID-19), and any potential misinformation must be promptly addressed. Scientific discourse is the basic foundation of science, and high-profile publications, especially controversial ones, require constructive dialogues for advancement of science and betterment of society, particularly during an ongoing war against a global pandemic.
